# Suicidal Ideation and Mental Health: The Moderating Effect of Coping Strategies in the Police Force

**DOI:** 10.3390/ijerph18158149

**Published:** 2021-08-01

**Authors:** Eloísa Guerrero-Barona, Mónica Guerrero-Molina, Maria José Chambel, Juan Manuel Moreno-Manso, Natalia Bueso-Izquierdo, Carlos Barbosa-Torres

**Affiliations:** 1 Department of Psychology, University of Extremadura, 06006 Badajoz, Spain; eloisa@unex.es (E.G.-B.); jmmanso@unex.es (J.M.M.-M.); nbueso@unex.es (N.B.-I.); carlosbarbosa@unex.es (C.B.-T.); 2 Faculdade de Psicologia, Universidade de Lisboa, 1649-013 Lisboa, Portugal; mjchambel@psicologia.ulisboa.pt

**Keywords:** suicidal ideation, mental health, depression, anxiety, coping strategies, police officers

## Abstract

The suicide rate in the police force (Fuerzas y Cuerpos de Seguridad-FFCCSS) is estimated to be greater than that of the general population. The objectives of this paper are to detect mental health problems, in particular depression and anxiety, and to analyze the moderating effect of coping strategies on the relation between mental health and suicide ideation in police officers. The Suicidal Behavior Questionnaire (SBQ-R), Beck’s Depression Inventory (BDI), the Anxiety Inventory (STAI), and the Brief Cope have all been used in the study. The sample consists of 98 Spanish police officers, of whom 91.8% were male. The results indicate that depression and anxiety can predict suicidal ideation. Nevertheless, it must be said that coping strategies do not have a moderating effect in the relation between mental health and suicidal ideation in this professional group.

## 1. Introduction

The suicide rate in the police force is estimated to be greater than that of the general population [1,2]. However, there is a series of methodological problems in their estimation: most police officers are male, belonging to an age range in which the suicide rate is greater [3], there is a lack of groups with which to make adequate comparisons [4], and there is the difficulty of equating the police force and the general population [5].

In addition, many suicides are classified as accidental or undetermined deaths because of the stigma attached to suicide [6]. Nevertheless, there are studies which show that suicide rates are greater in the police force than in the general population [7]; while others have found no differences once corrections have been made for demographic factors [5], and other studies have found lower rates [8]. Furthermore, suicide in the police force is considered a serious problem in need of further research.

### 1.1. Suicidal Ideation in the FFCCSS

Suicidal ideation refers to thoughts focused on the desire to end one’s own life, as well as to ideas and feelings that life no longer has any value. Such thoughts are recurrent and accompanied by a plan of how to die [9]. Suicidal ideation is a strong indicator of emotional distress and a strong predictor of attempted or actual suicide [10]. Studies concerning suicidal ideation in the general population estimate a prevalence of around 7.2% [11], while studies of the prevalence in the police force have variable rates, higher than or equal to those found in the general population: 13% [1]; 10.1% [2]; or 7.4% [12]. Nevertheless, there are studies that have found lower rates. Thus, Berg et al. [13] spoke of a prevalence of 6.4% for suicidal ideation in the police force in Norway, while Grassi et al. [7] demonstrated that the risk of suicide in the Italian police is at around 1.2%. In this sense, it is necessary to consider that differences may exist between small and large, urban and rural departments, as well as between police force of different countries, since the tasks they have to perform and the level of exposure to traumatic events (e.g., violence, natural catastrophes, or serious accidents) may be different.

The risk factors associated with suicide and the propensity toward suicide in the police force are similar to those in the general population. In this sense, economic and personal factors have been identified as mental health problems, including alcohol abuse [14] and marital or interpersonal relationship problems [5]. The strongest predictor of actual suicide is to have previously attempted it. However, there also exists specific factors associated with the work position, such as being exposed to traumatic situations, inadequate managerial practices, shift work, or being under disciplinary investigation [4]. Nevertheless, further research is needed to clarify the exact contribution of work-related factors [5].

### 1.2. Mental Health and the Predisposition to Suicide in the Police Force

Mental health problems are the main risk factor associated with suicide [15]. In Spain, police officers undergo training that, in addition to physical and theoretical tests, involves a psychological study that includes a personality test and a personal interview. This should, in theory, guarantee the absence of any serious psychological problems. However, this does not indicate that such problems may not develop later for various reasons. In this line, studies conducted with this occupational group confirm that it is a high risk profession for developing mental health problems; a profession in which worries about the stigma and the fear of a possible negative effect on the individual’s career also makes it less likely that police officers will seek psychological help [16].

Among the mental health problems associated with suicide in the police force is depression [10] as well as alcohol abuse, a deeply rooted cultural practice among police officers [1]. In a study conducted by Chopko et al. [10], in which suicidal ideation in 193 officers was studied, they found that of the considered predictors (alcohol abuse, post-traumatic stress disorders (PTSD), post-traumatic growth, and exposure to stressors, related or not to work, including exposure to traumatic situations), the existence of depression was the greatest predictor of suicide. In this sense, the patterns of sensorial processing, affective temperaments, and traumatic experiences can specifically characterize those persons with important affective disorders, as well as having a role to play in the prediction of suicidal ideation [17,18].

Depression is a common but serious disorder that affects one’s emotional state, has a great clinical and social relevance, and is also linked to increased mortality [19]. People suffering from depression present a sad or irritable state of mind and have difficulties enjoying those activities they previously enjoyed [20]. Among the symptoms of depression, the DSM-5 [20] includes one that refers to suicidal ideation. The relationship between depression and suicide in the general population is well established [21] and there are also studies that have found this same relationship in the police force [10].

The prevalence of an episode of depression in the adult general population in the United States of America (USA) has been estimated at around 10.4% [22] and in the general population of Spain at 3.67% [23]; while the prevalence of an episode of depression among police officers in the USA has been estimated at around 12% [24]. In the case of Spain, around 4% of members of the Civil Guard police division (considered part of the military) always feel depressed and 19% sometimes feel depressed, as a consequence of work related stress; while in the case of police officers, these percentages are 4% for always feeling depressed and around 11% for sometimes feeling depressed [25]. Nevertheless, differences have not been found between the clinical or subclinical levels of depression between police officers and workers in other professions [26].

The studies that have examined the causes of depression in police officers have found that such demographic factors as age, educational level, or years of service are not relevant; while gender, marital status, and organizational factors (workload, effort, or support) can have greater importance [27].

Anxiety, however, can be defined as a universal, adaptive response to a threat that can also become maladjusted [28] as anxiety related disorders are among the most common disorders that exist [20]. Anxiety is a psychological state of emotional and motivational type arising in situations that threaten the individual’s goals, and which depends on the anxiety trait and the stressful circumstances present at any particular moment [29]. The anxiety trait refers to a general and lasting disposition to respond to different situations with anxiety [30].

Studies concerning the anxiety trait and the state of anxiety among police officers have found that they present similar levels to the general population [31] and that their levels may be relevant at the time of carrying out their duties [32]. Works concerning the prevalence of anxiety disorders in police officers show low rates, but not many studies have handled predictive variables. Those that have handled this question found that demographic factors are not relevant when predicting the level of anxiety; while character traits, a high workload, and low levels of reward are all associated with higher levels of anxiety [27].

In the general population, a relationship has been found between anxiety disorders and suicidal ideation. Thus, Cougle et al. [33] found that panic disorder, post-traumatic stress disorder, social anxiety disorder, and generalized anxiety disorder are all related to a higher risk of suicidal ideation when other variables were controlled. Similarly, concerning the relationship between anxiety and suicide in police officers, Berg et al. [13] found that, among those factors related to Norwegian police officers having attempted suicide in the past, they had problems with anxiety.

### 1.3. Coping Strategies and Suicidal Ideation in the FFCCSS

The way people cope with stress is important in determining its impact on workers’ health [34]. Traditionally, coping has been defined as an effort to manage and overcome demands and critical events that pose a challenge, threat, harm, loss, or benefit to an individual [35]. As for coping strategies, these can be defined as perpetual or behavioral cognitive responses that are triggered in order to avoid, control, or deal with difficult or stressful situations [36]. Coping strategies are frequently divided into two large groups: active vs. passive strategies. Active strategies for coping with stress, also called approximation strategies or strategies aimed at action, refer to attempts to face the conflictive event, i.e., they indicate adaptive efforts to compensate for the stressful situation. Conversely, passive strategies, or strategies aimed at avoidance or not directed toward action, are an indicator of the individual’s degree of vulnerability, since the use of these strategies is usually less successful and refers to not directly facing the adversity or to evasion or denial [37].

In this sense, passive coping strategies are considered to be less adaptive and are linked to the development of such mental health problems as depression, while active strategies protect against the development of these same problems [38,39].

The studies that have related coping strategies to suicide have shown that suicide can be considered the least adaptive coping strategy. Research into these strategies in police officers reveals two common themes: the first is that alcohol is frequently used as a coping strategy and that the abuse of alcohol consumption is related to a greater suicidal ideation; the second is that there is a relationship between work-related stress and the existence of depression, which may hinder the mobilization of adequate coping strategies to face such demands in a satisfactory way, thus leading to the development of suicidal ideation [1]. In addition, as mentioned above, suicidal ideation is a risk factor for actual suicide attempts [11].

### 1.4. Objectives and Hypotheses of the Research

Previous studies have shown that there is a relationship between depression and suicidal ideation in police officers. Similarly, anxiety can also be a relevant variable, since the evidence suggests that it is bidirectionally related to depression and that it may increase emotional distress or the probability of developing PTSD. In addition, coping strategies moderate these relationships because they have an interactive effect [40,41]. Using inadequate coping strategies can also increase the effect of strain, thus causing a strong effect of depressive symptoms and anxiety on suicidal ideation in police officers. Similarly, adaptive and functional strategies can act as protective factors that act as a buffer against the effect of depressive symptoms and anxiety on suicidal ideation.

Based on results obtained in previous studies, while also considering the high rates of suicide and the urgent need to implement preventive and intervention programs, our general objective in this study is to describe mental health problems, in particular depression, anxiety, and suicidal ideation and coping strategies in the FFCCSS, and to analyze the moderating effect of coping strategies on the relationship between mental health and suicidal ideation in the police force. We formulated the following research hypotheses:

**Hypothesis** **1.** *Anxiety and depression are indicators of suicidal ideation.*

**Hypothesis** **2.** *The relation between mental health and suicidal ideation in police officers is moderated by the use of coping strategies, in such a way that this relationship is stronger when passive coping strategies are used and, on the contrary, weaker when active strategies are used.*

## 2. Materials and Methods

### 2.1. Participants

The selection of the participants was conducted using a non-probabilistic sample of convenience. The final sample consisted of 98 police officers belonging to the FFCCSS of Extremadura (Spain), of whom 91.8% were male. The age range was as follows: 41 to 50 years (48.5%); 31 to 40 years (27.6%); and 51 to 60 years (20.4%). Only 2% were over 61 years of age and only one subject was under 30 years of age. The majority were married or had a partner (73.5%), while 15.3% of the sample was divorced and 4.1% separated. Only 7.1% were single.

As for educational level, most of the participants had completed secondary school to the age of 18 years (Bachillerato) (45.9%) and 24.5% had university studies. Most of the participants were on the basic level of police officer (81.6%), while 18.4% were higher ranking officers.

Concerning experience as police officers, most had a certain amount of experience, as 82% had been in the police force for over 6 years in the same position. A total of 81.6% worked shifts, and 89.8% worked a minimum of one weekend every three months, while 44% worked between 5 and 7 weekends and 25.5% more than 8 weekends. The remaining 19.4% worked 4 weekends or less every three months.

Conversely, most of the participants (63.3%) worked between 2 and 4 nights every month, although an important percentage of the sample (19.4%) did not work nights, while 12.2% worked one night shift per month and 5.1% worked between 5 and 7 nights every month.

Finally, an important percentage of the sample ate a balanced diet (86.7), did not smoke (72.4%), consumed alcohol only occasionally (75.5%), and practiced sports (82.7%).

The only exclusion criterion of the study considered was that the participants should not be off work due to ill health.

### 2.2. Instruments

- Work & Sociodemographic Questionnaire. Data were gathered ad hoc concerning the gender, age, marital status, work situation, educational level, experience, and work category.
- Suicidal Behavior Questionnaire-Revised (SBQ-R) by Osman et al. [42]. This is a self-reporting measure of four items that evaluate four aspects related to suicide. One example is: “How probable is it that you may try to commit suicide one day?” The administration of the questionnaire takes approximately 5 min and the total range of scores is between 3 and 18 points, with higher scores reflecting a greater risk of suicidal behavior. A score of 7 or above is classified as risk of suicidal ideation, whereas scores below 7 are classified as non-suicidal. The questionnaire shows adequate psychometric properties in both internal consistency and reliability [42]. It also has good levels of reliability and validity in Spanish samples [43]. In our study, it obtained a Cronbach’s Alpha coefficient of 0.72.
- Beck’s Depression Inventory (BDI-abbreviated version) by Beck & Beck [44]. This has been used to evaluate the intensity of the depressive symptoms. It consists of 13 items that evaluate the negative mood, pessimism, weight loss, fatigue, suicidal thoughts, self-incrimination, and sensation of failure. The scores range from 0 to 39 and allow a classification of absence of or minimal depression (0–4), slight depression (5–7), moderate depression (8–15), and severe depression (>15) [45]. We use the Spanish version, which has been shown to have a good internal consistency and reliability [46]. In our study, it obtained a Cronbach’s Alpha of 0.91.
- State Trait Anxiety Inventory (STAI) Spielberger et al. [47]. This was used in its international version, in the forms Y1 and Y2 to evaluate anxiety. It consists of two scales of 20 items, each which measure two dimensions of anxiety: trait and state. The anxiety trait (AT) allows subjects to describe how they feel generally, while the anxiety state (AS) refers to how they feel in a more specific situation. The test is aimed at adults, the duration is between 15 and 20 min and can be applied collectively or individually. The scale of the responses is a Likert type scale, scoring 0 (almost never/not at all), 1 (a little/sometimes), 2 (fairly often/often), or 3 (a lot/almost always). The total score in each of the subscales is between 0 and 60 points, the highest scores corresponding to higher levels of anxiety. The informed reliability (Cronbach’s alpha) was 0.90 for anxiety trait (AT) and 0.94 for anxiety state (AS) [48]. In our study, the internal reliability coefficients were 0.87, for both the AT and the AS.
- Brief Cope Questionnaire, the Spanish version of the Carver Coping Scale (COPE). The coping strategies were evaluated using the brief version of the COPE adapted to Spanish [49]. The scale consists of 28 items grouped into 14 subscales divided into active (active coping, planning, positive reinterpretation, acceptance, humor, instrumental support, and use of emotional support) and passive strategies (self-distraction, denial, behavioral disconnection, relief, religion, use of substances, and self-blame). The items are posed in terms of the actions or thoughts used as ways of coping and each item has four possible options for answering (0: never; 1: occasionally, 2: most of the time; and 3: all the time), referring to the frequency with which the person performs an action or has a thought. The high scores indicate a more frequent use of this style or way of coping. One example of an active coping item is: “I concentrate my efforts on doing something about the situation I am in”; while an example of a passive coping item is: “I concentrate on work or other alternative activities to take my mind off things”. The reliability coefficients of the original subscales have values of Cronbach’s alpha between 0.50 and 0.90 [50], which indicate an adequate internal consistency. In our study, we obtained internal consistency coefficients of 0.91 and 0.70 for the active and passive coping strategies, respectively.

### 2.3. Procedure

We first contacted the police authorities to inform them of the study’s objectives and of its viability. Following the interest shown, they agreed to participate, and we examined together the procedure to follow in-data gathering. We decided upon administering the evaluation instruments through the Google Drive tool, Forms. Then, a police officer from Badajoz and a deputy director from Cáceres volunteered their collaboration, following instruction, to inform all the participants about the study and to send them the questionnaires online.

The participants were guaranteed anonymity and were asked to be sincere and to collaborate fully. They gave an informed consent as voluntary participants in the study. The participants agreed to complete the evaluation instruments. The application of the tools took approximately 15 min. Four months after the initial mailing, the definitive sample was obtained.

All the procedures performed in studies involving human participants were in accordance with the ethical standards of the institutional research committee. Ethical clearance was obtained from the Bioethics and Biosecurity Commission of the University of Extremadura (Ref. 185/2020).

### 2.4. Data Analysis

In accordance with the study’s objectives and based on the nature of the analyzed variables, a descriptive, correlation data analysis was conducted, as well as a mediation analysis. The considered confidence interval was set at 95%. The Statistical Package for the Social Sciences, SPSS, version 20, was used for the statistical treatment of the data.

The correlation analysis was performed to obtain a preliminary idea of the relationships between the variables being studied. Similarly, a regression analysis was used with the support of PROCESS software to analyze the moderating effect of the coping strategies on the relation between mental health and suicidal ideation in police officers.

## 3. Results

First, it is clear that the average scores of the police officers are below that of a risk of suicidal ideation (*M* = 6.22; *SD* = 1.7). Nevertheless, 29 police officers (29.6%) are within the group at risk of suicidal ideation (Table 1).

The results obtained concerning the level of depression (*M* = 3.62; *SD* = 4.91) allows us to state that 72.4% of the sample does not show depression (*n* = 71), 13.3% shows slight depression (*n* = 13), 10.2% shows moderate depression (*n* = 10) and 4.1% shows severe depression (*n* = 4).

As for anxiety, the scores are average in both anxiety state (*M* = 33.52; *SD* = 6.94) and anxiety trait (*M* = 32.99; *SD* = 7.43).

With respect to coping strategies, active coping (*M* = 30.59; *SD* = 9.3) is more prevalent than passive coping (*M* = 20.42; *SD* = 4.71). To be precise, an important percentage of the participants use active strategies related to acceptance of the circumstances (51%) and planning strategies of action aimed at dealing with stress (49%). At the same time, we can see a decrease in the use of passive coping strategies related to the consumption of alcohol or other substances in order to feel better or to stand the stress (1%).

The correlation analyses (Table 2) show that suicidal ideation has a positive correlation with depression (*r* = 0.676; *p* < 0.001), anxiety state (*r* = 0.437; *p* = 0.017), and anxiety trait (*r* = 0.550; *p* = 0.007); thus, the mental health of the police officers is related to suicidal ideation.

As for coping strategies, passive strategies have a positive correlation with suicidal ideation (*r* = 0.231; *p* = 0.022); thus, the greater the use of passive coping strategies, the greater the suicidal ideation among police officers.

In order to determine the extent to which mental health and coping strategies significantly influence suicidal ideation among police officers, we performed a moderation analysis (Table 3).

Depression is significantly and positively related to suicidal ideation among police officers (*b* = 0.21, 0.25; *p* < 0.001). However, active (*b* = −0.01; *p* = 0.078) and passive (*b* = −0.01; *p* = 0.230) coping strategies were not shown to be a significant moderator of the stated relationship.

Similarly, the anxiety state is related to suicidal ideation among police officers (*b* = 0.11; *p* < 0.001). Nevertheless, the moderating effect of active (*b* = −0.01; *p* = 0.645) and passive (*b* = 0.01; *p* = 0.445) coping strategies was not statistically significant upon interacting with the anxiety state in its relation to suicidal ideation.

Conversely, the results do allow us to state that the anxiety trait is also positively related to suicidal ideation (*b* = 0.12, 0.14; *p* = < 0.001), although the active (*b* = −0.01; *p* = 0.150) and passive (*b* = −0.01; *p* = 0.948) coping strategies do not seem to moderate this relationship.

There is, therefore, a relationship between mental health and suicidal ideation in police officers irrespective of the use of coping strategies.

## 4. Discussion

The results of this study demonstrate that depression and anxiety can predict suicidal ideation. However, the coping strategies do not have a moderating effect on the relationship between mental health and suicidal ideation in this professional group.

The literature considers that suicidal ideation is more prevalent in the police force than in the general population [12,51,52]. In our study, the estimated prevalence of suicidal ideation, planning, and attempted suicide for the sample agrees with that found by Berg et al. [13]. Despite the fact that the average scores demonstrate that the police officers are not within the subgroup at risk of suicidal ideation, we can state that an important percentage of these professionals show suicidal behavior patterns. Thus, the results obtained are seven times greater than the prevalence of suicidal ideation in the general Spanish population, as estimated by Gabilondo et al. [53] and that they are also higher than the prevalence in other countries [24].

Within the domain of perceived mental health, numerous prior works of research show that depression is one of the most prevalent problems within this professional collective, as the appearance of depressive symptoms is higher in police officers than in the general population [54,55,56,57]. Despite the fact that being exposed to critical, stressful incidents and routine work stress may predispose police officers toward depressive symptoms [58], the results obtained demonstrate that, in the sample, police officers showed minimal depression, consistent with the work of Tuohy et al. [59]. In this sense, Violanti et al. [52] highlighted the fact that female police officers are more prone to depression; thus, their prevalence may be overestimated under the characteristics described in the sample.

Conversely, there are several studies that show a high level of anxiety in the collective of police officers [32,55,59]. However, we can conclude that police officers mainly show average scores in anxiety state and anxiety trait; thus, our research allows us to corroborate the proposals of Acquadro-Maran et al. [60] and Williot & Blanchette [61], as they stated that a low percentage of police officers were characterized by a high level of anxiety.

As for the coping strategies used by the police officers, they mainly use strategies related to acceptance and planning a course of action to deal with circumstances. These conclusions agree with those of Acquadro-Maran et al. [60], Casado and Franco [62], and Rodríguez and Scharagrodsky [63]. Nevertheless, the findings of Arble et al. [64] are different, as they state that a particularly harmful manifestation in this collective is passive coping through the use of alcohol and other substances as a means to avoid the effects of stress.

Numerous studies conducted with police officers have suggested reasons for optimism concerning the use of active coping strategies, as they fulfil a protective function against the effects of adverse situations experienced while on duty and which enable them to cope with the stress in a rational, planned way, creating a better atmosphere at work and greater general wellbeing [12,58,63,65,66]. Similarly, previous works of research have demonstrated that the use of negative strategies favors the appearance of psychological symptoms, of which those of depression and anxiety were the most common, which would explain the low prevalence of this symptomatology in the sample. As stated by Jenkins et al. [56], not everyone exposed to stressful or traumatic events as part of their job develops psychological symptoms; this can be due to certain protective factors that contribute to their capacity to handle such situations and to recuperate from being exposed to negative situations in life, among which active coping strategies are one of the most important factors.

Based on these research results, the first hypothesis is supported. Depression and anxiety allow us to predict suicidal ideation and are thus good predictors in this professional collective. We thus conclude, in line with Rodríguez et al. [67], that suicidal ideation depends on factors related to mental health. In this sense, the significantly higher average for depression and anxiety is associated with a greater probability of suicidal ideation in police officers. This finding is consistent with other studies, such as those conducted by Gabilondo et al. [53] and Violanti et al. [52].

As for the second hypothesis, the suicidal ideation in police officers is not associated with the coping strategies used to deal with stress, as in Rothmann and Van Rensburg [68] but in contrast to Beehr, Johnson, & Nieva [69]. Pienaar et al. [12] show that police officers at risk of suicidal ideation have lower levels of active coping strategies, using passive strategies instead that are related to avoidance. These results led them to consider that disconnecting from negative occurrences at work or avoiding becoming involved in these events would predispose the police officers to suicidal ideation. However, the results of our study indicate that coping strategies are not moderators of the relationship between mental health and suicidal ideation.

Since the participants of the present research mostly use active coping strategies, our results may be related to those expressed by Acquadro-Maran et al. [60], Allison et al. [58] and Arble et al. [64], as active coping strategies act as a protective factor against depressive symptoms and thus may reduce suicidal ideation through their indirect influence on the mental health of the officers. As in Rodríguez and Scharagrodsky [63], the predominance of this style of coping among police officers may be related to the possibility of conducting their work while neutralizing their distress through acceptance and strategy planning, which allows them to cope with the said stress.

It is necessary to consider limitations of this study. First, the perceived health data, as well as the coping strategies and the suicidal ideation, were evaluated using self-reporting measures, which can potentially introduce bias, leading to a conservative estimation of the associations observed. Increasing the sample size will allow us to establish more accurate estimations of the prevalence and prediction of suicidal ideation [52]. In addition, this increase will be important as it will also augment the possibility of finding significant relations between variables, particularly those of moderation.

Similarly, the transversal design of the research limits our capacity to establish a temporal relation between the variables under study, as well as to generalize the results obtained for this professional collective to the general population. It will be convenient, in the future, to conduct a longitudinal study. As the sample is one of convenience, another limitation is related to the representativeness and the selection procedure. These limitations can be overcome in future work by using a probabilistic sample that includes randomness and qualitative measures, such as discussion groups. In future research, it will be desirable to analyze the influence of demographic and labor variables in order to explore their contribution to the prevalence of the symptomatology, as well as in the prediction of suicidal ideation. Another area to investigate is the stress factors at work, their consequences, and the lifestyle that can negatively affect both their physical and psychological health.

This study is of an exploratory nature. In the future, through a different research project, it can be possible to analyze the sensorial processing patterns and such variables as cognitive distortions or irrational beliefs and ideas that can explain the depressive symptomatology and the vulnerability to suicidal ideation.

As already mentioned, it is our desire to increase the sample size in future studies, as well as increasing the participation of the police officers. Furthermore, our aim is to involve mental health professionals to conduct a treatment program.

## 5. Conclusions

One of the most significant contributions of this work is the mental health analysis and the analysis of the police officers’ coping strategies, since, as stated by Álvarez et al. [70], the study of the effects of the profession on this collective in Spanish samples is relatively scarce.

As pointed out in the introduction, the mission of police officers is to preserve life and protect society’s goods. To this aim, police officers are called to resolve different problems on individual, family, and social levels. In so doing, they are exposed to many critically stressful incidents, including the risk of suffering serious injury and death. It is thus considered to be a profession characterized by high levels of stress that can have consequences for their physical and mental health [54,56,57,59,63,64]. Similarly, the conditions in which they work are not always optimal (low salary, long shifts, job insecurity, scarce resources, lack of communication, conflicts at work, excessive paperwork, etc.), which all lead to an increase in pressure working as a police officer [54,57,58,63,71].

In light of the above considerations, this professional collective has been considered, due to the regular and continuous exposition to high levels of stress, an ideal group for studying the variables that can allow us to predict suicidal ideation [65].

Although it is possible to find studies that analyze the mental health of the police force [56,72], as well as the coping strategies used [64,65,71] and the risk of suicidal ideation [55,67], research regarding predicting the risk of suicidal ideation in police officers has focused on the study of different dimensions of the personality, such as neuroticism and extraversion [12]. The current research recommends that, when intervention programs related to the prevention and control of suicidal behavior patterns in the police force are implemented, special attention should be paid and priority given to depressive symptomatology. As stated by Violanti et al. [52], there are few studies that have specifically examined depression as a factor associated with suicide in police officers, despite the evidence that this collective shows higher percentages of suicidal behavior patterns than the general population, and that depression allows us to predict the risk of suicidal ideation. However, we believe that the predictive capacity shown among the variables analyzed in this work can be used as evidence to design, develop, and implement preventive and intervention measures focused on encouraging personal resources and reducing depression and anxiety.

The relevance of the study of suicidal ideation lies within its consideration as the risk factor that can most accurately predict the probability of future suicide [67,73,74]. Thus, based on the results found, we agree with Pienaar et al. [12] in considering that suicidal ideation should be included as an indicator of psychological distress.

## Figures and Tables

**Table 1:** Distribution of the sample scores.

	Minimum	Maximum	*M*	*SD*
Suicidal ideation	5	14	6.22	1.7
Depression	0	24	3.62	4.91
Anxiety state	20	48	33.52	6.94
Anxiety trait	22	54	32.99	7.43
Active coping	14	51	30.59	9.3
Active	2	8	4.52	1.89
Planning	2	8	5.09	1.81
Positive reinterpretation	2	8	4.51	1.81
Acceptance	2	8	5.31	1.83
Humor	2	8	3.97	1.73
Instrumental support	2	8	3.8	1.59
Use of emotional support	2	7	3.37	1.44
Passive coping	14	34	20.42	4.71
Self-distraction	2	8	3.45	1.58
Denial	2	5	2.34	0.74
Behavioral disconnection	2	6	2.75	1.07
Relief	2	8	3.5	1.38
Religion	2	8	2.78	1.47
Use of substances	2	6	2.14	0.64
Self-blame	2	8	3.45	1.32

**Table 2:** Correlations between suicidal ideation and mental health and coping strategies.

	Suicidal Ideation
Depression	0.676 **
Anxiety state	0.437 **
Anxiety trait	0.550 **
Active coping strategies	−0.126
Passive coping strategies	0.231 *

Note: * *p* < 0.05; ** *p* < 0.001.

**Table 3:** Mediation analysis of suicidal ideation with respect to mental health and coping strategies.

Predictor variable	*B*	*SE*	*t*	*p*	LLCI	ULCI
DV: suicidal ideation (*R*^2^ = 0.48; *p* < 0.001)						
Depression	0.21	0.03	7.40	0.000	0.15	0.27
Active coping strategies	−0.16	0.01	−1.16	0.247	−0.43	0.01
Depression x active coping strategies	−0.01	0.01	−1.78	0.078	−0.01	0.01
DV: suicidal ideation (*R*^2^ = 0.46; *p* < 0.001)						
Depression	0.25	0.03	8.05	0.000	0.19	0.32
Passive coping strategies	−0.01	0.03	−0.42	0.672	−0.07	0.05
Depression x passive coping strategies	−0.01	0.01	−1.21	0.230	−0.02	0.01
DV: suicidal ideation (*R*^2^ = 0.21; *p* < 0.001)						
Anxiety state	0.11	0.02	4.76	0.000	0.06	0.15
Active coping strategies	−0.02	0.02	−1.40	0.164	−0.06	0.01
Anxiety state x active coping strategies	−0.01	0.01	−0.46	0.645	−0.01	0.01
DV: suicidal ideation (*R*^2^ = 0.20; *p* < 0.001)						
Anxiety state	0.11	0.03	4.11	0.000	0.05	0.16
Passive coping strategies	−0.01	0.04	−0.06	0.947	−0.09	0.09
Anxiety state x passive coping strategies	0.01	0.01	0.77	0.445	−0.01	0.01
DV: suicidal ideation (*R*^2^ = 0.33; *p* < 0.001)						
Anxiety trait	0.12	0.02	6.05	0.000	0.08	0.16
Active coping strategies	−0.02	0.01	−1.59	0.116	−0.05	0.01
Anxiety trait x active coping strategies	−0.01	0.01	−1.45	0.150	−0.01	0.01
DV: suicidal ideation (*R*^2^ = 0.31; *p* < 0.001)						
Anxiety trait	0.14	0.02	5.92	0.000	0.09	0.18
Passive coping strategies	−0.04	0.04	−0.95	0.342	−0.11	0.04
Anxiety trait x passive coping strategies	−0.01	0.01	−0.06	0.948	−0.01	0.01

Note: DV: dependent variable.
